# Retrospective analysis of cases of intraoperative awareness in a large multi-hospital health system reported in the early postoperative period

**DOI:** 10.1186/s12871-020-00974-3

**Published:** 2020-03-09

**Authors:** Amanda S. Deis, Michael P. Schnetz, James W. Ibinson, Keith M. Vogt

**Affiliations:** 1 Department of Anesthesiology & Perioperative Medicine, University of Pittsburgh Medical Center, Pittsburgh, USA; 2 Department of Anesthesiology & Perioperative Medicine, University of Pittsburgh School of Medicine, 3459 Fifth Avenue, UPMC Montefiore, Suite 467, Pittsburgh, PA 15213 USA; 3 Clinical and Translational Science Institute, University of Pittsburgh, Pittsburgh, USA; 4 Department of Anesthesiology, Surgical Service Line, Veterans Affairs Pittsburgh Healthcare System, Pittsburgh, USA; 5 Department of Bioengineering, Swanson School of Engineering, University of Pittsburgh, Pittsburgh, USA; 6 Center for the Neural Basis of Cognition, Pittsburgh, USA

**Keywords:** Intraoperative awareness, General anesthesia, Depth of consciousness monitoring, Processed electroencephalogram

## Abstract

**Background:**

Awareness with recall under general anesthesia remains a rare but important issue that warrants further study.

**Methods:**

We present a series of seven cases of awareness that were identified from provider-reported adverse event data from the electronic anesthesia records of 647,000 general anesthetics.

**Results:**

The low number of identified cases suggests an under-reporting bias. Themes that emerge from this small series can serve as important reminders to anesthesia providers to ensure delivery of an adequate anesthetic for each patient. Commonalities between a majority of our identified anesthetic awareness cases include: obesity, use of total intravenous anesthesia, use of neuromuscular blockade, and either a lack of processed electroencephalogram (EEG) monitoring or documented high depth of consciousness index values. An interesting phenomenon was observed in one case, where adequately-dosed anesthesia was delivered without technical issue, processed EEG monitoring was employed, and the index value suggested an adequate depth of consciousness throughout the case.

**Conclusions:**

Provider-reported adverse event data in the immediate post-operative period are likely insensitive for detecting cases of intraoperative awareness. Though causation cannot firmly be established from our data, themes identified in this series of cases of awareness with recall under general anesthesia provide important reminders for anesthesia providers to maintain vigilance in monitoring depth and dose of anesthesia, particularly with total intravenous anesthesia.

## Background

Accidental intraoperative awareness with recall (AWR) is the unanticipated explicit recollection of intraoperative events during anesthesia. Though important to understand and prevent, it is fortunately a rare event. In fact, the incidence estimates vary widely, likely due to methodology differences in identification of awareness events. Several randomized controlled trials with AWR as the primary endpoint have used structured interviews to detect awareness events and expert panel review to adjudicate possible cases of AWR [1–6]. Averaging across the data from these trials gives an incidence of 0.25% for definite AWR and an additional 0.32% of patients having possible AWR. This is corroborated by an incidence of 0.44% in a recent meta-analysis that included randomized trials focused on either anesthetic regimens or anesthetic depth monitors (but not necessarily specifically focused on AWR detection) [7].

Retrospectively identifying cases of AWR, when no structured patient interview has been conducted, is expected to have a lower sensitivity. This is illustrated by much lower calculated incidences of AWR within retrospective studies of varying methodology: 0.023% in a single-institution retrospective chart review [8] and 0.0051% in the United Kingdom’s 5th National Audit Project [9]. Thus, retrospective anlayses have limited sensitivity, and may detect AWR events at rates 10–100 times lower than propsective trials. Surveying practicioners seems equally insensitive, with rates of 0.0043% [10] and 0.0065% [11] reported.

Despite this low prevalence, AWR is a clinically important phenomenon to understand and prevent. The Psych-SOS study completed post-traumatic stress disorder (PTSD) assessments of patients from three major awareness prevention trials and found a 43% incidence of PTSD in those who experienced AWR, compared to 16% in a matched cohort without AWR [12]. Though the longevity of PTSD has not been studied in a large cohort of AWR patients, symptoms can last for many years after the event [13, 14]. Even without a PTSD diagnosis, many AWR patients experience some negative symptoms such as sleep disturbance, anxiety, fear, panic, depression, and inability to work [15–17].

The purpose of this investigation was to identify cases of AWR at our institution by a retrospective review of routinely collected adverse event data. We expected to find many AWR cases, based on the very large number of anesthetic records available for review. However, the small number of cases identified precluded a formal analysis. Our results are presented as a truncated AWR case series, focused on general anesthetics. Commonalities between these cases are discussed to highlight some important considerations to maintain vigilance in AWR prevention.

## Methods

Cases of AWR were collected from the Electronic Anesthesia Record (EAR) system of the University of Pittsburgh Medical Center. This is a large multi-hospital health system, with a mix of tertiary/academic centers and suburban hospitals that share a centralized EAR. The system-wide EAR was queried for all available electronic anesthesia records over the period 9/13/2010 to 1/12/2019. Patients with AWR were identified using quality improvement records attached to our EAR. In this system, providers voluntarily denote an adverse event flag (labelled “Intraoperative Recall”) any time before the EAR chart is finalized, which is typically after patient discharge from Post-Anesthesia Care Unit (PACU). Following identification of those anesthetics that had the adverse event flag for “Intraoperative Recall”, the patient charts were reviewed by the authors for data thought to be relevant to the awareness event. We limited the subsequent analysis to cases done under general anesthesia. Data abstracted included the surgery performed, anesthetics administered, patient characteristics, and the patient’s past medical and surgical histories. Written documentation was searched for descriptions of the character of the experiences reported by the patients during the awareness event. All authors reviewed the documentation available for each case and made a determination by consensus of the likelihood of an AWR event (rated as Probable, Possible or inconclusive). Additionally, cases were classified using the Michigan Awareness Classification Instrument [18]. Case durations were calculated using the induction and emergence times documented on the EAR. In the UPMC system, the BIS monitor (Medtronic/Covidien, Mansfield, MA, USA) is the processed electroencephalographic (EEG) monitoring device typically available in most anesthetizing locations. Age-adjusted minimum alveolar concentration (aaMAC) was calculated for any volatile agents used, using previously-established formulas [19]. For the purposes of describing patient body habitus, we classified body-mass index (BMI) as normal-range (BMI = 18.5–30), obese (BMI = 30–40), and morbidly obese (BMI > 40).

## Results

During the study period, 1,273,060 total anesthetic records were available for query. Only 10 cases were identified in which the “Intraoperative Recall” event flag was marked. Thus, the calculated incidence of AWR from this dataset, using this method of identification was only 0.00079%. Three of these cases were documented as sedation with monitored anesthesia care, and these were excluded from further analysis. The number of general anesthesia cases queried was 647,009, giving a calculated incidence of 1:92,429 (0.0011%) of AWR under general anesthesia in our EAR. We briefly describe the 7 identified cases of AWR during general anesthesia in a condensed case series below. A summary of key case details is provided in Table 1. To retain patient anonymity, no patient-specific information has been included.

**Table 1:** Summary of key characteristics of identified AWR cases done under general anesthesia

Case #	Surgery Type	Age (decade)	Gender	ASA PS	Premedication	Patient risk factors for AWR	Maintenance anesthetics used	Notes	Classification of Recall
1	Spine	30s	M	2	midazolam & fentanyl	none	Sevoflurane, remifentanil	Early lowering of anesthetic agent and discontinuation of remifentanil infusion. BIS not used.	Possible; Class 2
2	Head & Neck	70s	M	3	midazolam	morbid obesity	Sevoflurane	BIS not used	Possible; Class 1
3	Orthopedic	60s	F	3	midazolam	obesity	Sevoflurane	Sevoflurane < 0.5 aaMAC for much of case, with BIS < 60	Probable; Class 5D
4	General	30s	F	3	none	morbid obesity	TIVA with propofol, dexmedetomidine, & ketamine	BIS > 65 during entire case	Possible; Class 2
5	General (abdominal)	50s	F	3	midazolam	none	TIVA with propofol, dexmedetomidine, & ketamine	BIS < 47 for entire case	Possible; Class 2
6	General	60s	F	2	midazolam	none	TIVA with dexmedetomidine and remifentanil, then propofol	BIS > 60 for much of case	inconclusive
7	Plastic	20s	F	2	midazolam	obesity	TIVA with propofol, & dexmedetomidine & ketamine	IV infiltration occurred. BIS not used	inconclusive

Classification of recall event is based on concordance of authors. Each case is classified as Probable, Possible, or inconclusive, for the likelihood of a true AWR event. Further, the perceptions described are described using the Michigan Awareness Classification Instrument [18], as described in the text

Case 1 occurred in a male patient in his 30’s with American Society of Anesthesiologists (ASA) physical status (PS) II presenting for elective spine surgery. This patient had a normal-range BMI. The patient was premedicated with 50 mg of fentanyl and 2 mg of midazolam. General anesthesia was induced with 130 mg of propofol, and muscle paralysis initiated with 50 mg of rocuronium. Intermittent boluses of rocuronium were used to maintain paralysis. Maintenance of anesthesia was with sevoflurane, and the lowest concentration during the majority of the 3.5 h-long case was 1.77% (aaMAC = 1.11). However, the concentration decreased to 0.9 to 1.1% (aaMAC = 0.57–0.69) in the approximately 10 min prior to turning the patient supine. This volatile anesthetic was augmented with a remifentanil infusion at 0.1 to 0.2 mcg/kg/min, and this infusion was discontinued approximately 10 min prior to turning supine. A total of 200 mcg of fentanyl was given in divided doses after extubation, approximately 20 min after discontinuation of the remifentanil infusion. Mean arterial pressures (MAP) was maintained in the range 80 mmHg to 120 mmHg, but this required a phenylephrine infusion at rates up to 0.5 mcg/kg/min plus intermittent boluses of ephedrine. His heart was in sinus rhythm, rates ranging 45 to 80 beats per minute (bpm). Processed EEG monitoring was not used.

Case 2 occurred in a morbidly obese male patient in his 70’s, ASA-PS III, for elective head/neck surgery. He was premedicated with 50 mcg of fentanyl and 2 mg of midazolam. General anesthesia was induced with 200 mg of propofol and 100 mcg of fentanyl. Muscle paralysis was maintained with boluses of rocuronium. Maintenance of anesthesia was achieved with intermittent boluses of fentanyl and sevoflurane; the lowest end-tidal sevoflurane concentration was 1.45% (aaMAC = 0.98). Vitals were remarkable for atrial fibrillation, with heart rates between 60 to 95 bpm and no hypotension. Processed EEG monitoring was not used. Case duration was 2.5 h.

Case 3 occurred in an obese female patient in her 60’s, ASA-PS III, for orthopedic surgery following a traumatic fracture. She was premedicated with 2 mg of midazolam. General anesthesia was induced with 180 mg of propofol, and 100 mg of lidocaine was given. Muscle paralysis initiated with 190 mg of succinylcholine and 30 mg of rocuronium. Maintenance of anesthesia was achieved with sevoflurane, and end-tidal concentrations ranged from 0.3 to 1.3% (aaMAC = 0.19–0.82) during the case. The BIS™ monitor was applied, and the highest BIS index value recorded was 54.8. Case duration was 2 h. Vital signs during the case were heart rates between 55 to 75 bpm and MAP ranging from 95 mmHg to 115 mmHg, with minimal support by intermittent phenylephrine and ephedrine.

Case 4 occurred in a morbidly obese female patient in her 30’s, ASA-PS III. She presented for elective abdominal surgery and received no premedication. Anesthesia was induced with 200 mg of propofol, 16 mcg of dexmedetomidine, and 20 mg of ketamine. Muscle paralysis initiated with 160 mg of succinylcholine and 90 mg of rocuronium with intermittent boluses of rocuronium throughout the case to maintain paralysis. The patient had a history of difficult airway management but was uneventfully intubated with a Glidescope. Total intravenous anesthesia (TIVA) was maintained with three infusions: propofol ranging from 100 to 150 mcg/kg/min, with additional intermittent boluses; dexmedetomidine at 0.4 mcg/kg/hr; and ketamine at 0.2 mg/kg/hr. Processed EEG monitoring was utilized during the case, but not applied until 5 min after incision, which was about 30 min after induction. The BIS index ranged 65–75 in the first 30 min after application. At this time, a relief in hands-on providers occurred, and midazolam 2 mg was given. The highest recorded BIS index was 79.2, and this occurred during the middle portion of the case approximately 45 min after induction. The BIS index was > 65 for the majority of the surgical case. Vital signs were unremarkable with no support. Case duration was just over 2 h.

Case 5 occurred in a female patient in her 50’s, ASA-PS III, with normal range BMI. She presented for general surgery. She was premedicated with midazolam 2 mg. Anesthesia was induced with 150 mg of propofol followed by rocuronium 30 mg to facilitate tracheal intubation. TIVA was maintained using a propofol infusion with a basal rate of 100 mcg/kg/min, with intermittent boluses, dexmedetomidine infusion at 0.2 mcg/kg/hr and ketamine infusion at 0.2 mg/kg/hr. The highest documented BIS index was 46.6. Vitals signs were remarkable only for mild bradycardia, with heart rates in the 50’s pre-induction. She was normotensive throughout the two-hour case, with no support.

Case 6 occurred in a female patient in her 60’s, with ASA-PS II and normal-range BMI, who underwent an elective plastic surgery. Prior anesthesia complications included postoperative nausea and vomiting. She was premedicated with midazolam 2 mg. General anesthesia was induced with 50 mg of propofol and 50 mcg of fentanyl. Paralysis was maintained with intermittent boluses of rocuronium. Maintenance of anesthesia employed remifentanil at 0.2 to 0.6 mcg/kg/min and dexmedetomidine between 0.2 to 0.7 mcg/kg/hr. Approximately 20 min after surgical incision (and 40 min after induction) a propofol infusion at 50 mcg/kg/min was started, and the dose was subsequently increased to 75 mcg/kg/min for the last hour of the case. Vitals were unremarkable, with minimal intermittent doses of phenylephrine and ephedrine. The highest BIS index of 75.2 occurred near the start of surgery, about 15 min after induction. The patient was administered more fentanyl and propofol at that time, and the BIS index subsequently remained between 47 and 61 for the remainder of the two-hour case. Other than selection of the “Intraoperative Awareness” flag, there is no further documentation of awareness.

Case 7 occurred in an obese female patient in her 20’s, with ASA-PS III, who presented for elective plastic surgery. The patient was premedicated with 2 mg of midazolam. General anesthesia was induced with 200 mg of propofol and 100 mg of lidocaine was given. Muscle paralysis was initiated with 10 mg of vecuronium and maintained with intermittent boluses. Maintenance of anesthesia was attempted with a TIVA approach utilizing infusions of propofol at 75 mcg/kg/min, dexmedetomidine at 0.6 mcg/kg/hr and intermittent boluses of midazolam (6 mg additional given). Vitals were notable for MAP ranges between 70 mmHg to 110 mmHg, sinus rhythm with heart rates 75 bpm to 100 bpm. Processed EEG monitoring was utilized during the case and notable BIS index ranging from 68 to 75 in the 25 min following incision. Despite additional intravenous (IV) medications including another 2 mg of midazolam, 150 mg of propofol bolus, and increased infusion dose of propofol to 125 mcg/kg/hr, the BIS index remained elevated above 70. An additional 10 mg dose of vecuronium was given and did not result in loss of TOF. It was then recognized that her IV access had been lost. The patient was started immediately on sevoflurane at 3.5%. A right internal jugular line was placed for definitive access. Additional midazolam was given and prior maintenance TIVA was continued. Notably, the BIS index ranged 30 to 40 for the remainder of the two-hour case. The awareness flag was selected by the anesthesiologist with concern for possible awareness. There is no further documentation of AWR.

## Discussion

To our knowledge, this is the largest pool of cases systematically evaluated for adverse event data related to anesthetic AWR. Additionally, our use of electronic anesthesia records allowed for a more accurate assessment of important data including BIS index, end-tidal volatile concentration, and vitals, which would be inherently limited in any review of paper records, as demonstrated previously [20]. Even using conservative estimates for the incidences of AWR (0.01%), one might anticipate ~ 65 cases from a dataset of over 647,000 general anesthesia records. However, our actual results were an order of magnitude lower, consistent with lower estimates using some other non-prospective identification methodologies [9–11]. We strongly suspect that our health system’s EAR adverse event data significantly underestimates the true incidence of AWR in our patient population. Thus, our case cohort is a potentially biased sample of early-presenting AWR cases, and we recognize that our identified cases have significant limitations in their predictive ability for AWR. Nonetheless, we are presenting the series of seven general anesthetic cases identified and have recognized some themes that are worthy of discussion.

Most well-known risk factors for AWR are based on descriptive data or case reports [21, 22]. The occurrence of AWR during cardiac, obstetric, and trauma surgical cases, seems intuitive, as these are situations in which it is likely to deliver lower anesthetic doses. Some patient-related risk factors are also not surprising, as they would predispose to anesthetic-resistance, including obesity and chronic alcohol or sedative use. Notably, most of these case- or patient- related risk factors did not emerge in our cohort, except that 4/7 were obese. In fact, all but one (case 3) were elective patients admitted from home.

Several risk factors for AWR related to medication choices have been variably reported in the literature. Relevant to our case series, the use of neuromuscular blocking drugs can mask patient movement that would likely provide an early clinical sign of light anesthesia. Correlation between pharmacologic paralysis and AWR has been suggested [23, 24], as well as increased distress of AWR patients who were unable to move during the awareness event [22]. It is notable that paralysis was used in all seven of our identified cases, substantiating this correlation. The pre-induction administration of benzodiazepines intuitively should be protective against AWR, by providing amnesia. In some studies, their administration has been anti-correlated to AWR risk [25, 26]. However, similar to larger studies [27], our series demonstrate that AWR can certainly still occur despite benzodiazepine premedication, as these were part of the anesthetic in all but case 4.

The use of TIVA has been correlated to AWR [27]. It makes intuitive sense that the use of TIVA may increase the risk, for two reasons related to specific favorable properties of volatile anesthetics. First, end-tidal gas monitoring allows breath to breath measurement of the dose of anesthetic delivered. If used in combination with processed EEG monitoring, expired anesthetic concentration provides potentially synergistic information that can be used to ensure an adequate dose and depth of anesthesia. Second, IV failure can cause an occult disruption of anesthetic delivery, and this is much more likely to go unrecognized than a breathing circuit disconnect. Only one case of IV infiltration was identified within the cohort of general anesthetics. However, it is worthy of note that one of the three sedation cases with AWR that were identified by our initial query also had IV infiltration noted as the suspected etiology. This highlights the importance of particular vigilance in assuring IV patency during TIVA cases to avoid AWR. Further, It has been previously demonstrated that lower-dose propofol TIVA with neuromuscular blockade and alfentanil, with or without midazolam premedication, resulted in a high incidence of AWR [28]. A TIVA technique with propofol ≤100 mcg/kg/min was used in cases 4, 5 and 7. Though cases 5 and 7 employed midazolam premedication, the lower propofol dosing strategy may have contributed to AWR in these three cases.

Improper or no use of depth of consciousness monitoring could play a role in AWR. The utility of processed EEG monitoring in preventing awareness has been shown not superior to end-tidal anesthetic gas concentration alarms, but is better than clinical signs alone [27]. It is also worthy of note that the previously-cited study on AWR with propofol TIVA [28] relied on clinical assessment to titrate anesthetic depth. This could seem to suggest that processed EEG monitoring should be applied in TIVA cases, whenever possible. However, notably the ASA’s practice advisory only recommends that their use be considered on a case-by-case basis [21]. It does, however, stand to reason that, if a processed EEG monitor is employed, the anesthetic should be modified if index values are consistent with light anesthesia. In our series, cases 1 and 2 did not employ BIS monitoring, likely due to field avoidance concerns, but these cases did employ volatile anesthesia. Cases 4 and 6 (both TIVA) employed BIS monitoring, but index values were elevated ≥60 during most of the case. Though retrospective and anecdotal, this does raise the question as to whether additional or multimodal anesthetics would have both reduced the BIS index and/or prevented AWR. Case 3 illustrates the situation of reassuring BIS index, but a low anesthetic gas concentration. This suggests that clinicians should consider ensuring both adequate empiric anesthetic dose and reassuring depth of consciousness index values.

The timeline within the case in which AWR seemed to occur in our case series is also worthy of note. Cases 1, 4, and 5 document specific recall of events near the end of their surgical experiences. This serves as a reminder that AWR most often occurs with light anesthesia, and patients are most likely to experience light anesthesia during emergence. Providers must balance the desire for a timely wake-up against the risk of a patient becoming aware while still experiencing noxious stimulation. Though not suggested by events in our case series, this can also be an issue in cases with a prolonged time between IV induction and initiation of the maintenance anesthetic, as with difficult airway management. The administration of additional anesthetic should be considered during this initial period, as appropriate.

Finally, patients with a history of AWR are at 5-fold increased propensity for experiencing AWR again - even when they are enrolled in an AWR-prevention trial [29]. This seems to suggest that a subset of the population might show anesthetic resistance, even in the setting of reassuring depth of consciousness monitoring and clinical signs. This is illustrated by case 5, where the BIS index and vital signs were consistent with the appearance of adequate general anesthesia. An interesting population-based measure of this phenomenon is illustrated by the spread of data in the first figure of Aranake’s previous study on AWR [29]. The top of the figure shows many data points with BIS indices above 60, despite being in a range of age-adjusted MAC that should be clinically adequate to ensure unconsciousness and amnesia. This unusual discordance in EEG response to anesthetics may be an area for future neuroscience investigation, to provide better, more personalized, anesthetic care for patients.

### Limitations

This case series has several limitations. The retrospective nature of the study was not able to discern clinical decision-making details that may have been involved in each case, and this limits the ability to specifically determine causative factors for each AWR event. We also do not have long-term psychiatric follow-up documented for any of the patients. The design was also limited in ability to identify cases, relying on self-reporting by providers marking an event flag in the EAR. This inherently restricted the time window for identifying and reporting AWR to the immediate post-operative period. Previous studies have suggested that up to 2/3 of cases present only after PACU discharge [9]. These factors likely contributed to our lower incidence, compared to previous studies that employed longer follow-up periods in their methodology.

### Future directions

We are implementing system-wide provider education surrounding anesthetic awareness prevention to reduce the occurrence of these potentially devastating events, including highlighting some of the important themes suggested by these cases. We are also reviewing our event reporting system, in general, with an aim to improve the capture rate of AWR and other rare, but important adverse events. Finally, the lack of specificity in documentation surrounding this series of AWR events suggests the need for a structured form to be used when both collecting data and offering follow-up to patients after an AWR event.

## Conclusions

In a systematic, retrospective analysis of electronic anesthesia records, we have demonstrated that provider-reported adverse event data recorded in the immediate post-operative period is insensitive for detecting cases of intraoperative awareness. In the series of cases of awareness under general anesthesia that were identified, there are several important points for anesthesia providers to consider, including maintaining vigilance in monitoring both depth and dose of anesthesia, particularly with total intravenous anesthesia.

## Data Availability

All data generated or analyzed during this study are included in this published article.

## Abbreviations

ASA: American Society of Anesthesiologists
AWR: Awareness with recall
BMI: Body-mass index
bpm: beats per minute
EAR: Electronic anesthesia record
EEG: Electroencephalogram
IV: Intravenous
MAP: Mean arterial pressure
PACU: Post-anesthesia care unit
PS: Physical status
PTSD: Post-traumatic stress disorder
TIVA: Total intravenous anesthesia
